# 改良MINE方案治疗侵袭性NK细胞白血病和T细胞淋巴瘤继发噬血细胞综合征3例并文献复习

**DOI:** 10.3760/cma.j.cn121090-20231115-00261

**Published:** 2024-06

**Authors:** 迪 吴, 淼静 李, 瑶 李, 彤霞 逯, 璐瑶 付, 鹏程 贺

**Affiliations:** 1 西安交通大学第一附属医院血液内科，西安 710061 Department of Hematology, the First Affiliated Hospital of Xi'an Jiaotong University, Xi'an 710061, China; 2 核工业四一七医院血液肿瘤科，西安 710600 Department of Hematology and Oncology, Nuclear Industry 417 Hospital, Xi'an 710600, China

## Abstract

淋巴瘤相关噬血细胞综合征病情凶险，进展迅速，特别在NK/T细胞淋巴瘤多见。MINE方案为侵袭性非霍奇金淋巴瘤挽救性治疗方案。西安交通大学第一附属医院应用改良MINE方案治疗3例继发于侵袭性NK细胞白血病和T细胞淋巴瘤的噬血细胞综合征患者，初步显示该方案对NK/T细胞淋巴瘤的疗效以及对继发噬血细胞综合征炎症状态的控制和良好的耐受性。

## Abstract (en)

Lymphoma-associated hemophagocytic syndrome is aggressive with rapid progression, particularly in NK/T cell lymphoma. The MINE regimen is a salvage treatment for aggressive non-Hodgkin lymphoma. In our center, the modified MINE regimen was applied to treat three patients with hemophagocytic syndrome secondary to aggressive NK cell leukemia and T-cell lymphoma. The modified MINE regimen showed good efficacy against NK/T cell lymphoma, control of the inflammatory state of secondary hemophagocytic syndrome, and good tolerability.

噬血细胞综合征又称噬血细胞性淋巴组织细胞增多症（HLH），是一种由基因突变或感染、炎症或肿瘤触发的危及生命的免疫过度激活状态[1]。HLH病情发展迅速，死亡率较高。淋巴瘤相关HLH病情更复杂，预后较差，在治疗上仍存在巨大挑战。既往研究显示MINE（美司钠+异环磷酰胺+米托蒽醌+依托泊苷）方案对复发或难治性侵袭性非霍奇金淋巴瘤（NHL）患者有效，且耐受性良好，但MINE方案用于治疗NK/T细胞淋巴瘤相关HLH尚缺乏相关报道。近期，本中心应用改良MINE方案治疗了3例继发于侵袭性NK细胞白血病和T细胞淋巴瘤的HLH患者，其中以米托蒽醌脂质体替换原方案中米托蒽醌，具体方案为：异环磷酰胺0.5～1 g/m^2^、米托蒽醌脂质体10 mg、依托泊苷50～75 mg/m^2^，第1天至第3天。3例患者临床初步疗效报告如下并进行文献复习。

## 病例资料

例1，男，65岁，反复高热2周。实验室检查：WBC 1.05×10^9^/L、ANC 0.36×10^9^/L、HGB 64 g/L、PLT 14×10^9^/L、ALT 131 U/L、AST 250 U/L、血清铁蛋白37 244.00 ng/ml、纤维蛋白原0.59 g/L、甘油三酯4.37 mmol/L、NK细胞活性25.05％（参考区间≥15.11％）、sCD25>50 000 U/ml（参考区间223～710 U/ml）以及EBV-DNA 5.19×10^5^拷贝。超声提示脾脏厚径44 mm，长径107 mm。全身CT：颈部双侧多发小淋巴结；双肺下叶后基底段慢性炎症伴条索，右肺中叶膨胀不良；纵隔未见明显肿大淋巴结；肝脏多发囊肿，胆囊多发结石。骨髓穿刺涂片：不明细胞占56％。骨髓活检原位组化：淋巴瘤，需鉴别NK细胞肿瘤。免疫组化：CD19^−^、CD20^−^、CD3^+^、CD5^−^、CD2部分^＋^、CD7^−^、CD56^+^、CD4^−^、CD8^−^及GrB^+^。染色体核型分析：可见克隆性异常add（2q）、del（7q）、−8、add（13q）、add（14p）、−15、−17、add（17p）、der（18）、+20、add（21p）、+mar1及+mar2。血液肿瘤相关基因检测：T淋巴瘤克隆性基因重排检测阳性，B细胞淋巴瘤克隆性基因重排检测阴性。诊断：①HLH；②侵袭性NK细胞白血病。2022年9月1日开始MINE方案诱导化疗，每日输注血制品持续1周，维持HGB>70 g/L，PLT>30×10^9^/L。化疗结束后3 d，WBC最低降至0.24×10^9^/L，ANC降至0，血清铁蛋白5 582 ng/ml EBV-DNA 4.29×10^3^拷贝，纤维蛋白原自行上升，>1.0 g/L。化疗前和粒缺期应用亚胺培南抗感染11 d，降钙素原由1.41 ng/ml降至正常，化疗第2天体温正常，后未再出现反复。2022年9月15日三系细胞逐渐恢复，粒细胞缺乏恢复，WBC 1.44×10^9^/L、ANC 0.74×10^9^/L、HGB 89 g/L、PLT 40×10^9^/L，好转后出院。化疗结束3周后复查指标：WBC 5.24×10^9^/L、ANC 3.68×10^9^/L、HGB 87 g/L、PLT 352×10^9^/L、ALT 14 U/L、AST 13 U/L、血清铁蛋白2 496.00 ng/ml，纤维蛋白原5.02 g/L，EBV-DNA 1.12×10^3^拷贝。复查骨髓涂片：侵袭性NK细胞白血病完全缓解骨髓象。微小残留病（MRD）：异常细胞约占有核细胞的0.32％（3.2×10^−3^）。行MINE方案联合培门冬酶（3 750U）巩固化疗1个疗程，化疗结束后11 d，WBC最低降至0.98×10^9^/L，ANC 0.19×10^9^/L，应用粒细胞集落刺激因子3 d后粒细胞缺乏恢复。复查骨髓涂片：侵袭性NK细胞白血病完全缓解骨髓象。骨髓流式细胞术检测MRD阴性。EBV-DNA阴性。行鞘内注射2次。于2022年11月28日接受半相合（女供父）异基因造血干细胞移植。移植后1年，监测骨髓：NK细胞白血病移植后完全缓解骨髓象。骨髓流式细胞术检测MRD阴性，病情稳定。

例2，女，38岁，间断发热9个月余。2022年9月因外周血三系细胞减少伴发热，体温反复波动于38～39 °C，于当地医院诊断为T细胞大颗粒淋巴细胞白血病，予以口服环孢素治疗。仍有发热，停用环孢素，经伏立康唑抗感染后体温恢复正常，三系细胞逐渐升至正常。再次复查骨髓涂片、免疫分型和骨髓活检原位组化未发现异常细胞，未进一步治疗。2023年6月再次出现高热，伴三系细胞进行性下降。以“发热待查”住我院感染科，实验室检查：WBC 1.50×10^9^/L，ANC 0.79×10^9^/L，HGB 76 g/L，PLT 9×10^9^/L，血清铁蛋白4 379.00 ng/ml，纤维蛋白原1.66 g/L，甘油三酯4.56 mmol/L，NK细胞活性1.3％（提示NK细胞活性减低），sCD25计数为37 410 U/ml，EB病毒定量阴性。超声提示脾脏厚径49 mm，长径143 mm。全身CT：双肺上叶后段及下叶背侧胸膜下间质性病变并条索灶，双侧胸膜增厚；纵隔未见明显肿大淋巴结；脾大。给予地塞米松及人免疫球蛋白冲击治疗2周后，三系细胞均未回升。骨髓结果回报：约17％不典型淋巴细胞（倾向肿瘤性），可见少量噬血现象。骨髓活检原位组化：T细胞淋巴瘤侵犯骨髓，基因重排TCR阳性。染色体核型分析：47,XX,+20[1]/46,XX[3]。因患者血小板降至0，外出风险大，未行PET-CT检查。诊断：①HLH；②T细胞淋巴瘤Ⅳb期（IPI评分3分），转入血液科。2023年7月13日血常规WBC 0.43×10^9^/L，ANC 0.08×10^9^/L，HGB 81g/L，PLT 0×10^9^/L。每日输注血小板基础上开始给予MINE方案诱导化疗，化疗前和粒缺期应用亚胺培南抗感染14 d，监测降钙素原正常，化疗期间患者体温逐渐降至正常，未出现明显出血倾向，2023年8月10日粒细胞缺乏恢复，WBC 1.30×10^9^/L、ANC 0.53×10^9^/L、HGB 83 g/L，PLT 95×10^9^/L。甘油三酯1.59 mmol/L，血清铁蛋白3 862 ng/ml。复查骨髓涂片：增生性骨髓象。流式细胞术检测检测MRD阴性。予第2个周期MINE方案化疗，复查骨髓涂片：增生性骨髓象，流式细胞术MRD阴性。骨髓原位组化未见异常，基因重排TCR阴性。PET-CT：全身未见葡萄糖代谢异常增高灶。予第3个周期MINE方案化疗，行鞘内注射1次。后2次化疗均未出现粒缺和严重骨髓受抑，PLT最低降至37×10^9^/L。2023年11月28日行自体造血干细胞移植，目前随诊中。

例3，女，40岁。间断高热伴皮疹2个月余，2023年6月2日来我院，体温波动于37.5～40 °C，面部、胸背部及四肢皮肤可见密集红色皮疹，实验室检查：WBC 2.56×10^9^/L，ANC 1.59×10^9^/L，HGB 75 g/L，PLT 91×10^9^/L，ALT 118 U/L，AST 100 U/L，血清铁蛋白36 758.00 ng/ml，纤维蛋白原4.34 g/L，甘油三酯3.87 mmol/L。NK细胞活性3.5％（提示NK细胞活性减低），sCD25计数为2 362 U/ml，EB病毒定量阴性。全身PET-CT：①双侧颈动脉鞘周围、双侧颈根部、双侧锁骨上窝、双侧腋窝、腹主动脉旁、双侧腹股沟区多发肿大淋巴结且葡萄糖代谢增高，骨髓葡萄糖代谢轻度增高，符合淋巴瘤，多维尔评分4分。②肝大，肝囊肿；脾大。骨髓涂片：增生性骨髓象。免疫分型未见明显异常。颈部淋巴结活检病理结果考虑外周T细胞淋巴瘤，非特指型。诊断：①HLH；②外周T细胞淋巴瘤，非特指型，Ⅲb期（IPI评分2分）。2023年6月13日行MINE方案化疗，未出现粒缺和严重骨髓抑制，PLT最低降至52×10^9^/L。化疗后第2天体温降至37.9 °C，化疗结束后5 d体温正常，皮疹消褪。复查WBC 3.16×10^9^/L、ANC 2.71×10^9^/L、HGB 74 g/L、PLT 108×10^9^/L、ALT 56 U/L、AST 31 U/L及血清铁蛋白1 153.00 ng/ml。患者回当地医院继续CHOP（环磷酰胺+阿霉素+长春新碱+泼尼松）方案化疗，随诊病情稳定，疾病缓解状态。

## 讨论及文献复习

HLH是一种由异常活化的巨噬细胞和细胞毒性T细胞所致的严重的炎症反应综合征[2]。临床以持续发热、全血细胞减少、肝脾肿大为主要表现，骨髓、淋巴结和肝脾组织中可见巨噬细胞吞噬血细胞现象。淋巴瘤是导致继发HLH的重要病因之一[3]。淋巴瘤诊疗过程中发生的HLH统称为淋巴瘤相关HLH（LA-HLH），其中，NK/T细胞淋巴瘤占所有恶性肿瘤相关HLH比例为35.2％[4]。未及时诊断及治疗的LA-HLH预后差，中位生存时间短于2个月[5]。因此，早期诊断、分层治疗及充分的器官功能支持，才能为患者后续治疗创造条件[6]。

LA-HLH患者的治疗包括针对HLH的诱导治疗和针对淋巴瘤的原发病治疗。目前研究显示[7]，对于脏器功能较差的患者，诱导治疗推荐HLH-94方案，HLH得到控制后过渡到淋巴瘤的治疗。对于器官功能尚可的患者，可给予兼顾HLH及淋巴瘤的联合化疗方案，如DEP（脂质体阿霉素+依托泊苷+甲泼尼龙）、Ru-DEP（芦可替尼+DEP）或Ru-DED（芦可替尼+脂质体阿霉素+依托泊苷+地塞米松）、L-DEP（L-天冬酰胺酶+DEP）、和DA-EPOCH（依托泊苷+阿霉素+长春新碱+环磷酰胺+泼尼松）方案。一项关于DA-EPOCH方案治疗淋巴瘤相关HLH的临床研究显示[8]，该方案对于B细胞淋巴瘤相关HLH患者的疗效较好，但无法改善NK/T细胞淋巴瘤相关HLH患者的预后，因此仍需探索针对NK/T细胞淋巴瘤相关HLH更为有效的治疗方案。近年来，随着对HLH发病机制的深入研究，新型靶向药物，如依帕伐单抗[9]、芦可替尼[10]、阿仑单抗[11]等对复发/难治性LA-HLH患者显示出初步疗效，目前正进一步开展相应的临床试验，以期改善这部分患者的生存预后。

针对LA-HLH的方案仍然存在残留病灶清除不彻底、HLH易复发，骨髓抑制严重、感染风险较大的问题。MINE方案具有兼顾治疗HLH和NHL的有效成分。Rodriguez等[12]报道MINE方案治疗48例复发耐药的NHL患者，结果显示，治疗有效率为48％，完全缓解率为21％。共随访51个月，中位生存时间为9个月，MINE方案的主要不良反应为骨髓抑制。Fan等[13]也报道了MINE方案治疗38例复发或耐药的侵袭性淋巴瘤的观察性研究，结果显示MINE方案的主要优点为患者耐受性高，主要问题为缓解时间较短。米托蒽醌脂质体通过对活性成分米托蒽醌进行脂质体包裹后，改变了药物在体内的药代动力学特征以及组织分布，显示出更高的抗肿瘤效果和更低的毒性。Ⅱ期研究结果表明，在复发/难治外周T细胞淋巴瘤患者中，米托蒽醌脂质体单药客观缓解率达到41.7％（95％ *CI* 32.3％～51.5％），且对蒽环天然耐药的NK/T细胞淋巴瘤疗效显著[14]。关于MINE方案治疗LA-HLH尚未见报道，我们尝试以米托蒽醌脂质体替代米托蒽醌的改良MINE方案用于治疗继发于NK/T细胞淋巴瘤相关的HLH，通过提高原发病疗效，迅速减轻炎症反应、加深HLH的缓解深度和降低复发率。

本研究3例HLH患者，其中1例继发于侵袭性NK细胞白血病且EBV定量阳性，2例继发于T细胞淋巴瘤，以改良MINE方案诱导化疗，化疗期间体温降至正常，炎症指标及脏器功能迅速改善。2例患者原发病起源于骨髓，在经过1个疗程MINE方案诱导化疗后达到完全缓解，其中1例达到MRD阴性。初步显示了该方案对侵袭性NK细胞白血病和T细胞淋巴瘤的疗效以及对继发HLH炎症状态的控制。3例患者均未因化疗导致严重感染及出血，表现出对该方案有良好的耐受性。改良MINE方案还需要不断探索，开展前瞻性临床研究进一步验证疗效。
